# An Evaluation of the Properties of Urethane Dimethacrylate-Based Dental Resins

**DOI:** 10.3390/ma14112727

**Published:** 2021-05-21

**Authors:** Agata Szczesio-Wlodarczyk, Monika Domarecka, Karolina Kopacz, Jerzy Sokolowski, Kinga Bociong

**Affiliations:** 1 University Laboratory of Materials Research, Medical University of Lodz, ul. Pomorska 251, 92-213 Lodz, Poland; 2 Department of General Dentistry, Medical University of Lodz, ul. Pomorska 251, 92-213 Lodz, Poland; monika.domarecka@umed.lodz.pl (M.D.); jerzy.sokolowski@umed.lodz.pl (J.S.); kinga.bociong@umed.lodz.pl (K.B.); 3 “DynamoLab” Academic Laboratory of Movement and Human Physical Performance, Medical University of Lodz, ul. Pomorska 251, 92-216 Lodz, Poland; kkopacz@mum.edu.pl; 4 Department of Health Sciences, Medical University of Mazovia, Ludwika Rydygiera 8, 01-793 Warszawa, Poland

**Keywords:** dental resins, UDMA, Bis-GMA, Bis-EMA, TEGDMA, mechanical properties, hardness, water absorption, water dissolution

## Abstract

Most of the dental materials available on the market are still based on traditional monomers such as bisphenol A-glycidyl methacrylate (Bis-GMA), urethane dimethacrylate (UDMA), triethyleneglycol dimethacrylate (TEGDMA), and ethoxylated bisphenol-A dimethacrylate (Bis-EMA). The interactions that arise in the monomer mixture and the characteristics of the resulting polymer network are the most important factors, which define the final properties of dental materials. The use of three different monomers in proper proportions may create a strong polymer matrix. In this paper, fourteen resin materials, based on urethane dimethacrylate with different co-monomers such as Bis-GMA or Bis-EMA, were evaluated. TEGDMA was used as the diluting monomer. The flexural strength (FS), diametral tensile strength (DTS), and hardness (HV) were determined. The impacts of material composition on the water absorption and dissolution were evaluated as well. The highest FS was 89.5 MPa, while the lowest was 69.7 MPa. The median DTS for the tested materials was found to range from 20 to 30 MPa. The hardness of the tested materials ranged from 14 to 16 HV. UDMA/TEGDMA matrices were characterized by the highest adsorption values. The overall results indicated that changes in the materials’ properties are not strictly proportional to the material’s compositional changes. The matrices showed good properties when the composite contained an equal mixture of Bis-GMA/Bis-EMA and UDMA or the content of the UDMA monomer was higher.

## 1. Introduction

One of the most important dental achievements in the last century was the introduction of resin matrix composites as a restoration material [1,2]. The organic matrix is typically based on dimethacrylate resins [3,4,5], while fillers primarily consist of silicon, quartz, borosilicates, zirconium, and aluminum oxides. Inorganic components have different sizes, shapes, and morphologies [6,7]. In dentistry, rear restorations (class I or II according to Black [7]) require composites that have good mechanical properties, while frontal restorations (classes IV and V) require excellent aesthetics. Thus far, no dental composite that meets all these requirements has appeared on the market [2]. Therefore, there is great interest in identifying dental composites with improved esthetics, and antibacterial, physical, and mechanical properties.

The most commonly used monomer in dental composites is bisphenol A-glycidyl methacrylate resin (Bis-GMA). Due to its high viscosity, which is caused by strong intermolecular interactions and the formation of hydrogen bonds between macromolecules, low-viscosity monomers are needed to dilute the polymer matrix and obtain desirable properties [8]. Typical diluent substances include dimethacrylate monomers such as triethylene glycol dimethacrylate (TEGDMA), ethylene glycol dimethacrylate (EGDMA), ethylene diglycol dimethacrylate (DEGDMA), 2-hydroxyethyl methacrylate (HEMA), and 1,10-decanediol dimethacrylate (DDDMA or D3MA) [9]. Free-radical chain polymerization of the organic phase, most commonly initiated by photo initiators or by a chemical initiator and co-initiator, leads to the formation of a cross-linked network bound by esters, urethanes, amide bonds, and van der Waals interactions [10]. Table 1 summarizes the selected properties of popular dimethacrylate homopolymers [11,12].

The aim of the present study was to determine the flexural strength (FS), diametral tensile strength (DTS), and hardness (HV) of polymer matrices based on urethane dimethacrylate (UDMA) with TEGDMA, Bis-GMA, and ethoxylated bisphenol-A dimethacrylate (Bis-EMA) as co-monomers. The dynamic water absorbency was also studied. The null hypothesis was: there is no effect of compositional changes in more complex resin systems on the three-point bending flexural strength (TPB), diametral tensile strength (DTS), Vickers hardness (HV), and the dynamics of water absorbency of the materials.

## 2. Materials and Methods

In this study, flexural strength, diametral tensile strength, and hardness were determined. Due to teeth anatomy and the nature of jaw mechanics, loads applied at restoration will cause various stresses, e.g., tensile and shear stresses. Therefore, it is justified to conduct a broader evaluation of mechanical properties, which will determine the behavior of the material under challenging mechanical conditions. Tensile loading is considered the most appropriate. However, it is a difficult test to conduct for dental materials. Diametral tensile strength is proposed as a substitute method. During this test (DTS) tensile, compressive and shear stresses are developed. A flexural strength test is recommended by ISO 4049 [13] for all restorative materials. The hardness is easy to test and may indicate some wear resistance of the materials [14,15]. High water absorption values may indicate that the material will be more susceptible to hydrolytic degradation [16].

Before mechanical tests, samples were immersed in water and stored at 37 °C for 24 h. Such a protocol is in accordance with ISO 4049 [13]. In order to ensure the completion of the post-cure polymerization processes, an interval of 24 h from the preparation of samples was used [17]. The impact of material composition on the water absorption and dissolution was evaluated.

### 2.1. Materials

The monomers used in the study are described in Table 2. Fourteen different resin mixtures were prepared according to the weight percentage of selected monomers (Table 3). Each mixture contained camphorquinone (an initiator (CAS 10373-78-1), <1 wt.%) and N,N-dimethylaminoethyl methacrylate (CAS 2867-47-2). After mixing, the resins were stored for a week prior to the study. The materials were cured for 20 s. Increments of 2 mm in thickness were polymerized. To ensure consistent irradiance values, the light curing units (Mini L.E.D, Satelec, France) were calibrated with a radiometer system (Digital Light Meter 200, Rolence Enterprice Inc., Taoyuan, Taiwan).

### 2.2. Flexural Strength

**Figure A1:**
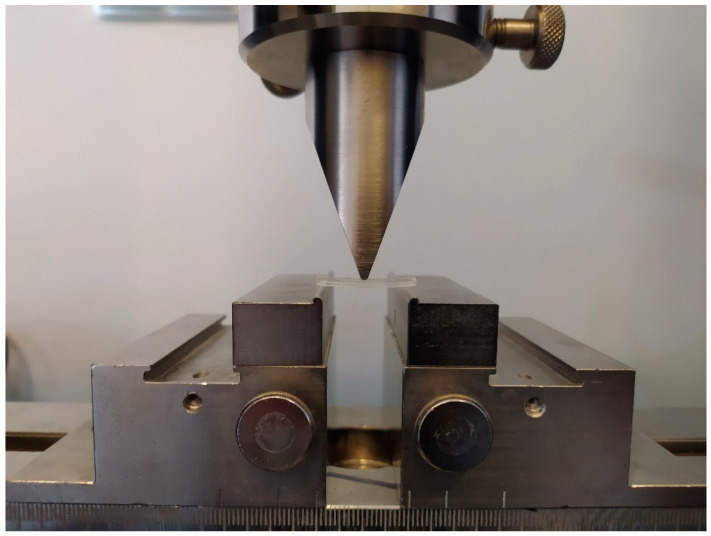
The three-point bending test.

Flexural strength (FS) was determined using the three-point bending test (Appendix A, Figure A1). Rectangular samples (dimensions: 2 mm × 2 mm × 25 mm) were used for the tests. For each study group, seven samples were tested. Measurements were carried out using a Zwick Roell Z020 universal strength machine (Zwick-Roell, Ulm, Germany). The traverse speed was 1 mm/min. During the test, the modulus of elasticity in bending was also determined.

### 2.3. Diametral Tensile Strength

**Figure A2:**
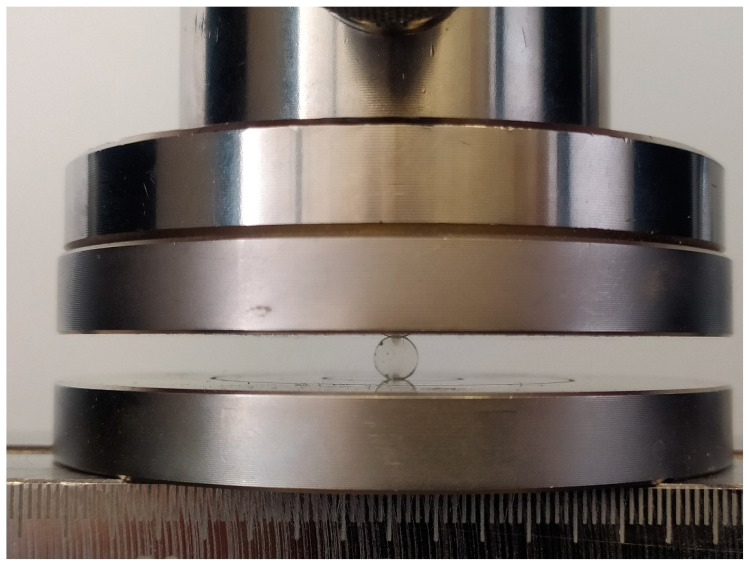
Diametral tensile strength test.

The tests were performed on samples in the form of a cylinder (6 mm in diameter and 3 mm in height) (Appendix A, Figure A2). The DTS was measured on nine samples from each study group using a Zwick Roell Z020 universal strength machine (Zwick-Roell, Ulm, Germany). The traverse speed was 2 mm/min. The DTS values were calculated using Equation (1):

(1) $$DTS=\frac{2F}{\pi dh}\;\left(MPa\right)$$

*F*—force that caused the destruction of the sample [N],
*d*—diameter of the sample [mm],
*h*—height of the sample [mm].

### 2.4. Hardness

**Figure A3:**
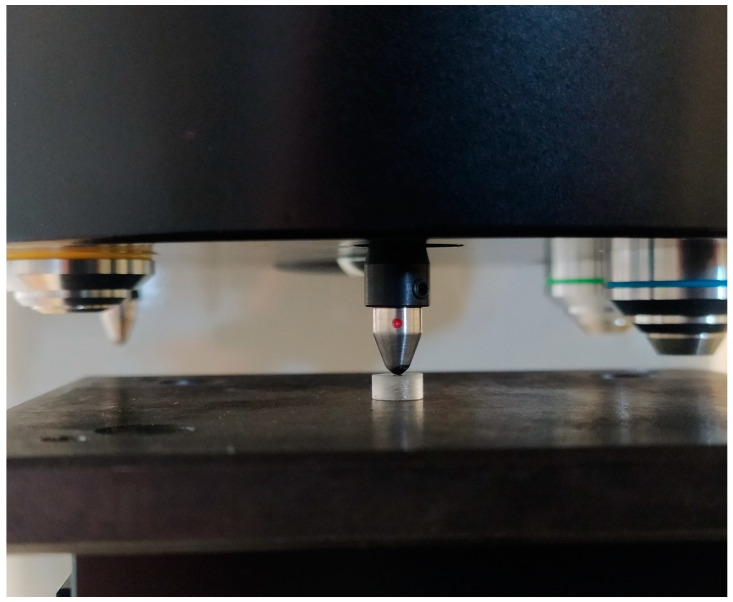
Vickers hardness test.

The hardness of the tested materials was measured using the Vickers method using a Zwick ZHV2-m hardness tester (Zwick-Roell, Ulm, Germany) (Appendix A, Figure A3). The applied load was 1000 g and the penetration time was 10 s. Nine measurements were performed on three out of nine DTS samples for each study group.

### 2.5. Dynamic Absorbency

In order to determine the dynamic absorbency, the samples were prepared using a silicone mold (15 mm in diameter and 1 mm in width). Tested materials were applied in one layer and cured with an LED light lamp (Mini L.E.D., Acteon, Norwich, France) in nine zones partially overlapping, in accordance with ISO 4049 recommendations [13]. Five samples were prepared for each dental composite. The samples were weighed (RADWAG AS 160/C/2, Poland) immediately after preparation, on 30 consecutive days, and then after 60, 90, and 120 days. The absorbency was calculated according to Equation (2):

(2) $$A=\frac{m_{i}-m_{0}}{m_{0}}\times 100\%$$

A—the absorbency of water,
*m*_0_—the initial mass of the sample,
*m*_i_—the mass of the sample after storage in water for a specified (*i*) period of time.

After 120 days, the specimens were dried to a constant weight using a protocol similar to the dissolution test from standard 4049 [13]. The weight loss (dissolution) in water was calculated according to Equation (3), this being the absolute value:

(3) $$D=\left|\frac{m_{0}-m_{z}}{m_{0}}\times 100\%\right|$$

*D*—the dissolution in water,
*m*_0_—the initial mass of the sample,
*m*_z_—the constant mass of the sample after drying.

### 2.6. Statistical Analysis

The obtained data were processed with the use of Statistica 13.1 (Statsoft, Kraków, Poland). For statistical analysis, elements of descriptive statistics were used. The Shapiro–Wilk test was used to confirm normality. As the data were found to be nonconsistent with a normal distribution, the data were then analyzed using the Kruskal–Wallis test with the multiple comparisons of mean ranks. The accepted level of significance was α = 0.05.

## 3. Results

**Figure 1:**
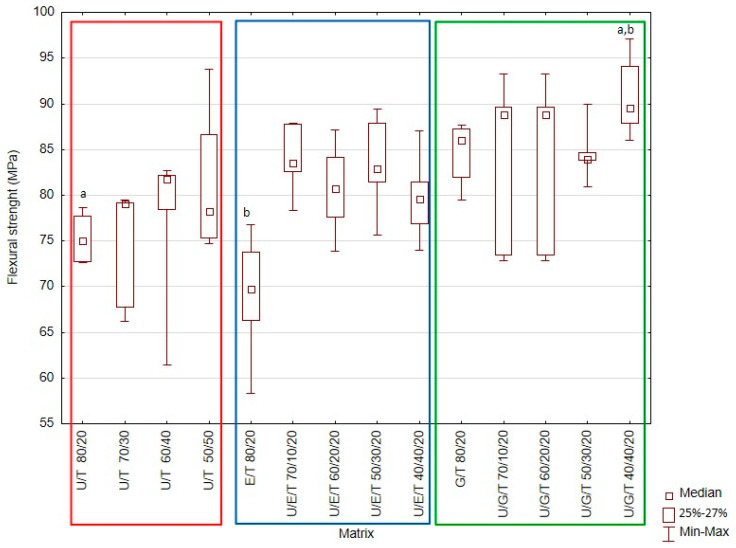
Box-and-whisker plot of three-point bending flexural strength (FS). For variables with the same letter (a,b), the difference is statistically significant (*p* ≤ 0.05).

**Figure 2:**
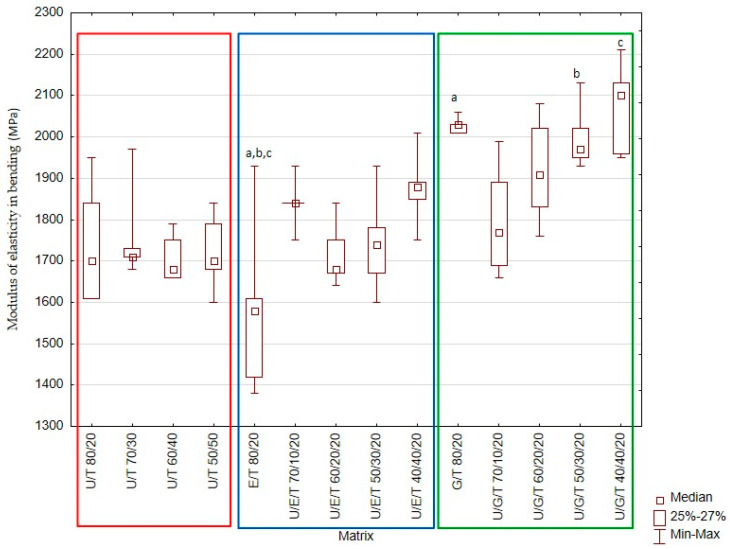
Box-and-whisker plot of modulus of elasticity in bending. For variables with the same letter (a,b,c), the difference is statistically significant (*p* ≤ 0.05).The modulus of elasticity in bending (FM) was also determined (Figure 2). Median FM values ranged from 1.58 GPa (Bis-EMA/TEGDMA 80/20 wt.%) to 2.1 GPa (UDMA/Bis-GMA/TEGDMA 40/40/20 wt.%) (Figure 2). These differences were statistically significant (Kruskal–Wallis test; *p*-value = 0.0000). Based on the multiple comparisons of mean ranks for all groups, statistically significant differences were also found between Bis-EMA/TEGDMA 80/20 wt.% and Bis-GMA/TEGDMA 80/20 wt.% (*p*-value = 0.010261); Bis-EMA/TEGDMA 80/20 wt.% and UDMA/Bis-GMA/TEGDMA 50/30/20 wt.% (*p*-value = 0.044215); Bis-EMA/TEGDMA 80/20 wt.% and UDMA/Bis-GMA/TEGDMA 40/40/20 wt.% (*p*-value = 0.044215).

**Figure 3:**
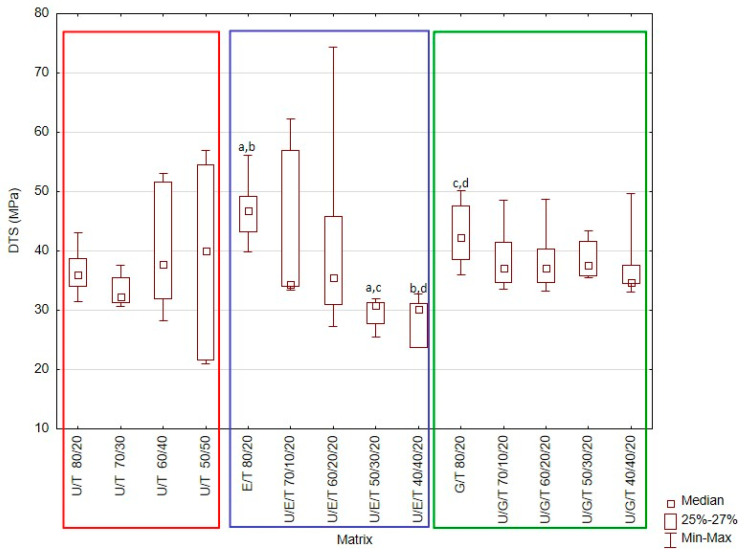
Box-and-whisker plot of diametral tensile strength (DTS). For variables with the same letter (a–d), the difference is statistically significant (*p* ≤ 0.05).

**Figure 4:**
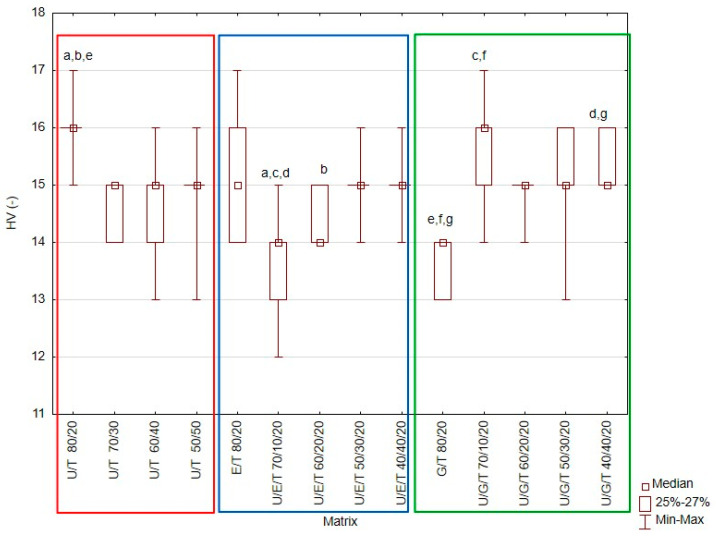
Box-and-whisker plot of Vickers hardness (HV) of tested materials. For variables with the same letter (a–g), the difference is statistically significant (*p* ≤ 0.05).

**Figure 5:**
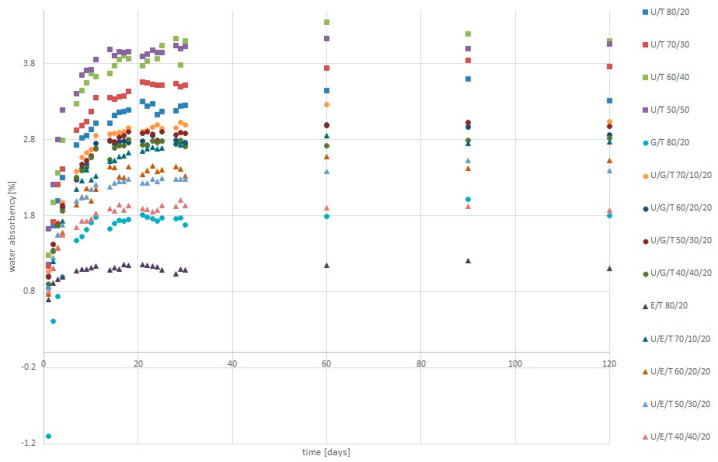
Dynamic water absorbency of tested matrices.

The obtained results are presented in Figure 1, Figure 2, Figure 3, Figure 4 and Figure 5 and Table 4.

### 3.1. Flexural Strength

The highest median value of the three-point flexural strength was 89.5 MPa (UDMA/Bis-GMA/TEGDMA 40/40/20 wt.%), while the lowest was 69.7 MPa (Bis-EMA/TEGDMA 80/20 wt.%) (Figure 1). These values were significantly different (Kruskal–Wallis test; *p*-value = 0.0042). Based on the multiple comparisons of mean ranks for all groups, statistically significant differences were found between UDMA/Bis-GMA/TEGDMA 40/40/20 wt.% and UDMA/TEGDMA 80/20 wt.% (*p*-value = 0.044215); UDMA/Bis-GMA/TEGDMA 40/40/20 wt.% and Bis-EMA/TEGDMA 80/20 wt.% (*p*-value = 0.04118). UDMA/Bis-GMA/TEGDMA 40/40/20 wt.% demonstrated a higher FS value than UDMA/TEGDMA 80/20 wt.% or Bis-EMA/TEGDMA 80/20 wt.% did. An analysis of the median FS depending on the matrix composition is presented in Figure 1.

In the case of Bis-EMA/TEGDMA 80/20 wt.%, the FM value was lower than those in Bis-GMA/TEGDMA 80/20 wt.%, UDMA/Bis-GMA/TEGDMA 50/30/20 wt.%, and UDMA/Bis-GMA/TEGDMA 40/40/20 wt.%. The analysis of the median modulus of elasticity in bending, depending on the matrix composition, is presented in Figure 2.

### 3.2. Diametral Tensile Strength

Median DTS values ranged from 30.1 MPa (UDMA/Bis-EMA/TEGDMA 40/40/20 wt.%.) to 46.8 MPa (Bis-EMA/TEGDMA 80/20 wt.%.). This difference was statistically significant (Kruskal–Wallis test; *p*-value = 0.0001). Based on the multiple comparisons of mean ranks for all groups, statistically significant differences were also found between: Bis-EMA/TEGDMA 80/20 wt.%. and UDMA/Bis-EMA/TEGDMA 50/30/20 wt.% (*p*-value = 0.001177); Bis-EMA/TEGDMA 80/20 wt.%. and UDMA/Bis-EMA/TEGDMA 40/40/20 wt.% (*p*-value = 0.000668); Bis-GMA/TEGDMA 80/20 wt.%. and UDMA/Bis-EMA/TEGDMA 50/30/20 wt.% (*p*-value = 0.013824); Bis-GMA/TEGDMA 80/20 wt.%. and UDMA/Bis-EMA/TEGDMA 40/40/20 wt.% (*p*-value = 0.008395).

Bis-EMA/TEGDMA 80/20 wt.% and Bis-GMA/TEGDMA 80/20 wt.% demonstrated higher DTS values than UDMA/Bis-EMA/TEGDMA 50/30/20 wt.% and UDMA/Bis-EMA/TEGDMA 40/40/20 wt.% did. An analysis of the median DTS with regard to material is presented in Figure 3.

### 3.3. Hardness

The highest median Vickers hardness (HV) value was 16 (-) (UDMA/TEGDMA 80/20 wt.% and UDMA/Bis-GMA/TEGDMA 70/10/20 wt.%), while the lowest was 14 (-) (UDMA/Bis-EMA/TEGDMA 70/10/20 wt.% and Bis-GMA/TEGDMA 80/20 wt.%.) (Figure 4). These differences were statistically significant (Kruskal–Wallis test; *p*-value = 0.0000). Most of the tested resin matrices had a hardness of 15. Based on the multiple comparisons of mean ranks for all groups, statistically significant differences were found between: UDMA/TEGDMA 80/20 wt.% vs. UDMA/Bis-EMA/TEGDMA 70/10/20 wt.% (*p*-value = 0.000292); UDMA/TEGDMA 80/20 wt.% vs. UDMA/Bis-EMA/TEGDMA 60/20/20 wt.% (*p*-value = 0.022403); UDMA/Bis-EMA/TEGDMA 70/10/20 wt.% vs. UDMA/Bis-GMA/TEGDMA 70/10/20 wt.% (*p*-value = 0.011175); UDMA/Bis-EMA/TEGDMA 70/10/20 wt.% vs. UDMA/Bis-GMA/TEGDMA 40/40/20 wt.% (*p*-value = 0.014966); Bis-GMA/TEGDMA 80/20 wt.% vs. UDMA/TEGDMA 80/20 wt.% (*p*-value = 0.000081); Bis-GMA/TEGDMA 80/20 wt.%. vs. UDMA/Bis-GMA/TEGDMA 70/10/20 wt.% (*p*-value = 0.003782); Bis-GMA/TEGDMA 80/20 wt.%. vs. UDMA/Bis-GMA/TEGDMA 40/40/20 wt.% (*p*-value = 0.005057).

### 3.4. Water Absorbency Dynamic Study

The matrices based on UDMA and TEGDMA had the highest absorbency. Bis-EMA-TEGDMA 80/20 wt.% showed the lowest water absorbency (Figure 5, Table 4). The highest dissolution values were observed for matrices with Bis-GMA (Table 4).

## 4. Discussion

The properties of the polymer matrix depend on its composition. The most popular base monomer used in dental composites is Bis-GMA, with a molecular weight (MW) of 512 g/mol; the compound comprises a stiff bisphenol A core and hydroxyl groups that are able to form strong hydrogen bonds [18]. Hence, it demonstrates a high viscosity (1200 Pa·s) [12]. An alternative base monomer with a flexible aliphatic core, and, hence, a lower viscosity (23 Pa·s), is UDMA (MW = 470 g/mol) [12]. It also has two urethane links, which are able to form hydrogen bonds, but these interactions are not as strong as in Bis-GMA. Still, this hydrogen bond, formed by the urethane proton donor group, is strong enough to increase the mechanical properties of dental composites [19]. Due to the more flexible nature, UDMA demonstrates a higher degree of conversion than Bis-GMA does and a higher morphological homogeneity [19,20,21].

Bis-EMA has a similar structure to Bis-GMA, being based on a stiff bisphenol A core; however, as it lacks two pendant hydroxyl groups and has longer ethoxylated linkages, Bis-EMA (MW = 540 g/mol) is more flexible and mobile, with a lower viscosity (0.9 Pa·s) than Bis-GMA [12]. Therefore, it demonstrates a higher overall conversion [12,22]. Finally, TEGDMA (MW = 286 g/mol) is a flexible, low-viscosity (0.01 Pa·s) diluent monomer [12], which is used to obtain a higher degree of conversion and filler homogenization. However, it is characterized by a high hydrophilicity and greater susceptibility to cyclization and polymerization shrinkage [23].

It is important to underline that the final properties result not only from the characteristics of individual monomers, but above all, from the interactions that arise in the monomer mixture and the characteristics of the resulting polymer network [24]. Recent research has examined new monomers based on methacrylate, urethanes, or new resin systems [19,20,25,26,27,28,29]. However, only a few commercial materials, such as Venus Diamond (Heraeus Kulzer), Kalore (GC Corp), and SDR (Dentsply Sirona), are based on such modified monomers [30,31]. Most of the restoration materials on the market are based on conventional resins (Bis-GMA, TEGDMA, UDMA, and Bis-EMA) [29]. The most extensively researched resins are Bis-GMA/TEGDMA and UDMA/TEGDMA and, more rarely, Bis-EMA/TEGDMA mixtures [12,18,22,23,32,33,34,35,36,37,38,39].

The present study examined the effect of the addition of TEGDMA, Bis-GMA, and Bis-EMA on selected properties of the matrix based on UDMA resin. The null hypothesis can be rejected due to changes in the evaluated properties, along with the modification of resins composition. Increasing the amount of TEGDMA monomer in the U/T mixtures did not affect the modulus of elasticity. However, it resulted in a slight increase in the flexural strength (FS) value, but this was not statistically significant; this may be due to the polymer network demonstrating a greater conversion and crosslink density [32]. Studies have shown that the addition of TEGDMA reduces the viscosity of systems based on Bis-GMA or UDMA, thus allowing a higher degree of conversion. However, excessively high amounts of diluent monomer (TEGDMA) result in the deterioration of properties of the tested matrices probably due to primary cyclization. The FS values for mixtures based on a molar fraction of UDMA or Bis-GMA of approx. 0.7 were approximately 140 MPa for a molar fraction of TEGDMA of approximately 0.3, but close to 110 MPa for a molar fraction of TEGDMA of more than 0.6 [32]. Lower-viscosity resins are more likely to demonstrate TEGDMA primary cyclization. Cyclization leads to a reduction in the effective cross-linking density and heterogeneity in the polymer due to microgel formation [38]. Composites based on Bis-GMA and 66% TEGDMA were characterized by high conversion values, but lower shrinkage values than would be expected, probably due to more severe primary cyclization. Although primary cyclization increases the conversion, it can compromise network formation and reduce the crosslinking density [39]. It is possible to achieve a high conversion with a relatively low TEGDMA content in UDMA-based composites (compared to Bis-GMA), due to their lower viscosity and structure characteristics [36]. A compromise between the degree of conversion and the desired properties (leaching and mechanical strength) was achieved for the systems with base monomers, Bis-GMA or UDMA, with molar fractions between 0.375 and 0.625 [32].

The addition of Bis-GMA in U/B/T mixtures was found to increase the flexural modulus, which has an impact on the value of the three-point bending strength (Figure 1 and Figure 2). The highest median values (FS = 89.5 MPa) were observed for the UDMA/Bis-GMA/TEGDMA 40/40/20 wt.% matrix, while the lowest FS (69.7 MPa) and FM (1.6 GPa) values were demonstrated by the Bis-EMA/TEGDMA 80/20 wt.% matrix. The addition of UDMA had a positive effect on the tested properties, with the resulting polymer network being characterized by a greater stiffness and resistance to three-point bending. The addition of Bis-GMA and UDMA in the mixtures U/G T and U/E/T, respectively, increased intermolecular interactions, mostly hydrogen bonding, which is considered to be one of the most important factors influencing the strength and modulus of crosslinked dimethacrylate systems [11,24,40]. High values of FS in mixtures containing Bis-EMA may indicate a high conversion. This monomer is less viscous than Bis-GMA and the systems can therefore achieve a higher degree of conversion [41]. Additionally, the introduction of UDMA to the U/E/T mixtures resulted in the formation of a denser polymer network. UDMA resins have a higher reactivity than Bis-EMA resins due to the greater flexibility of their molecular structure, possible hydrogen abstraction, and chain transfer reaction mechanism [18,22]. The FS values of unfilled resins based on UDMA/TEGDMA ranged from 44 to 78 MPa, while those of matrices based on Bis-GMA/TEGDMA were between 51 and 66 MPa [37]. The ISO 4049 demands a flexural strength of at least 80 MPa for restorative materials in occlusion-bearing areas. Resins obtained in this study will allow the requirements of the standard to be met after using a filler system.

The second most frequently defined mechanical property for polymeric dental materials is diametral tensile strength (DTS). This property allows the tensile strength to be indirectly examined [42]. This value for dental composites varies from 30 to 55 MPa [43]. However, it should be emphasized that the DTS value increases with filler content. The DTS of unfilled Bis-GMA/TEGDMA (75 wt.%/25 wt.%) resin was previously found to be 21.9 MPa [44]. The median DTS for tested materials was found to range from 20 to 30 MPa, with the highest value being observed for the Bis-EMA/TEGDMA 80/20 wt.% matrix (approximately 46 MPa). The DTS test assumes a negligible deformation before fracture. Some distortions may have appeared in the tests on resin matrices as they demonstrate greater plastic deformation than filled materials [33,45]. The materials containing Bis-EMA and TEGDMA tended to display greater deformations. In addition, the E/T 80/20 wt.% matrix had the lowest FM and modulus of elasticity, which suggests that it should also have a lower DTS value. The smallest dispersion of values was observed for the U/G/T matrices, which may be due to the formation of a stiffer and brittle system that was more suitable for this type of test.

Of the studied properties, hardness is one of the most sensitive to changes in the degree of conversion [46]. This value increases with the degree of conversion [47]. In the present study, the highest hardness value was demonstrated by the UDMA/TEGDMA 80/20 wt.% matrix (16 HV) and the lowest by the UDMA/Bis-EMA/TEGDMA 70/10/20 wt.% and Bis-GMA/TEGDMA 80/20 wt.% matrices (14 HV) (Figure 4). Previous studies have indicated that U/T matrices have higher hardness values than U/G matrices do; UDMA has a lower viscosity and is more flexible than Bis-GMA, which leads to a higher conversion and denser polymer network [37]. However, like other properties of dental resins, hardness also depends on intramolecular interactions and the polymer structure [24]. The presence of aromatic rings and urethane bonds increases hardness values [48].

When dental resins are soaked in water and oral fluids, unreacted monomers and small oligomers are eluted, and water is absorbed by the resin matrix. The absorbed water occupies the space between polymer chains or it is bonded with the polymer. This process is controlled by diffusion and requires a few weeks to complete [49,50]. Our dynamic absorbency testing showed that the fastest mass increase due to water sorption occurs during the first month (Figure 5). The UDMA/TEGDMA mixtures showed a more rapid increase than UDMA/Bis-EMA/TEGDMA. In addition, the highest values were observed for the U/T and U/G/T mixtures (Table 4).

Hydrophobicity of the monomer is one of the most important factors that allows water sorption to be predicted. In our study, the highest values of water absorbency were observed for U/T. The values did not change significantly within the selected formulations. High values were also observed for mixtures with Bis-GMA. Due to the presence of urethane linkages in UDMA, ether linkages in TEGDMA, and hydroxyl groups in Bis-GMA, monomers have a hydrophilic nature and will more easily cause water to penetrate into the polymer network. The smallest values of water absorbency were observed by formulations with the Bis-EMA monomer. This monomer decreases water sorption and solubility due to its hydrophobic character [11,51]. An additional factor that significantly affects sorption and solubility values is the degree of conversion, and the characteristic of the polymer network. Homogenous networks with high cross-linking densities and small levels of porosity or microvoids have reduced solvent uptake and swelling [50,52,53,54]. This factor may explain the higher sorption values for U/T matrices than for formulations with the addition of a more hydrophilic monomer such as Bis-GMA. The higher addition of TEGDMA in the U/T matrices could result in a more cross-linked network, which may create a more heterogeneous polymer structure [35]. Additionally, the structure may be disturbed by the occurrence of a cyclization process of TEGDMA monomer [18]. The more heterogeneous the structure, the larger the spaces created between the polymer clusters (microporous), which can accommodate a larger amount of water [35]. The high sorption values for U/T matrices can be explained by the higher flexibility of the network in comparison with U/B/T formulations (Figure 2). This permits the higher swelling of polymer chains by water [35]. Solubility and sorption cause the hygroscopic expansion, plasticization, and hydrolytic degradation of resins, thus weakening the mechanical properties over time [16]. Therefore, knowledge of the behavior of the resin under the influence of the aquatic environment is also a very important element in assessing its properties.

Little research has been performed into more complex resin systems. One of the most extensive works was published in 1998 by Asmussen and Peutzfeldt, but in this study, the resins were loaded with silanized glass filler (78 wt.%) [33]. Blends based on TEGDMA/UDMA/Bis-GMA showed good properties, when the composite contained an equal mixture of Bis-GMA and UDMA or the content of UDMA monomer was higher. Flexural strength values were found to be 159 ± 18, 164 ± 18, and 167 ± 12 MPa for composites based on TEGDMA/UDMA/Bis-GMA with monomer contents (mol%) of 30/40/30, 30/30/40, and 30/20/50, respectively. The modulus values for these materials were 10.2 ± 0.4, 9.1 ± 1.2, and 8.0 ± 0.7 GPa, respectively. The tensile strength was similar for all matrices (approximately 55 MPa) [33]. Similar studies found that medium-viscosity resin (TEGDMA/UDMA/Bis-GMA 30:33:33 wt.%) provided optimum mechanical properties, and that the viscosity should be adjusted to achieve a balance between efficient conversion and the best mechanical properties [34]. Another similar study based on only five experimental groups also suggested that Bis-GMA:TEGDMA:UDMA (30:35:35 mol%) resin showed promising properties [55].

It should be noted that this work had some limitations. A fairly wide spectrum of tests were carried out that allowed for the exclusion of matrices, which did not meet certain strength criteria. However, for a more complete evaluation, the viscosity of the formulations, the degree of conversion, and the polymer network structure should be determined. Currently selected formulations (both unfilled and filled systems) are under evaluation using the aging protocol to assess the behavior of these materials in complex oral environments.

## 5. Conclusions

Certain relationships were observed regarding the influence of individual components on the properties of polymer matrices. However, they are not directly proportional to the compositional changes. Matrices with compositions of UDMA/Bis-GMA/TEGDMA 70/10/20 wt.% and 40/40/20 wt.%, and UDMA/Bis-EMA/TEGDMA 40/40/20 wt.% matrices are characterized with a good flexural strength (FS), modulus of elasticity (ME), hardness (HV), diametral tensile strength (DTS), and satisfactory water absorption and dissolution values. The use of three different monomers in proper proportions may create a stronger polymer matrix. Tested formulations after filling should meet the requirements of standard 4049 on the minimum flexural strength for restoration materials. The DTS and hardness values were also promising.

In addition to the degree of conversion—which can be partially controlled by obtaining medium-viscous systems—an important issue is secondary bonds such as hydrogen bonding and van der Waals forces. These interactions may improve the mechanical properties by increasing the polymer network density. The addition of such monomers as UDMA and Bis-GMA, which, due to their chemical structure, are capable of producing such interactions, may result in the creation of a material with a higher strength.

## Figures and Tables

**Table 1:** Selected properties of A-glycidyl methacrylate (Bis-GMA), triethyleneglycol dimethacrylate (TEGDMA), urethane dimethacrylate (UDMA), and ethoxylated bisphenol-A dimethacrylate (Bis-EMA)—the most popular monomers used in dental composites.

Monomer	Molecular Weight (g/mol)	Viscosity (Pa·s)	Flexural Strength (MPa)	Flexural Modulus (GPa)	Water Sorption (µg/mm^3^)	Solubility (µg/mm^3^)
Bis-GMA	512	1200 ^a^	72.4 ^b^	1 ^b^	51.2 ^b^	9.5 ^b^
TEGDMA	286	0.01 ^a^	99.1 ^b^	1.7 ^b^	28.8 ^b^	27.5 ^b^
UDMA	470	23 ^a^	133.8 ^b^	1.8 ^b^	42.3 ^b^	20.4 ^b^
Bis-EMA	540	0.9 ^a^	87.3 ^b^	1.1 ^b^	21.3 ^b^	2.1 ^b^

^a^—taken from [12]; ^b^—taken from [11].

**Table 2:** Monomers used in the study.

Monomer	Abbreviation	Manufacturer	Purity	Viscosity at 25 °C
Bis-GMA	G	Esstech, Inc., Essington, PA, USA	97%	718,641 cps
TEGDMA	T		99.8%	---
UDMA	U		98.4%	9387 cps
Bis-EMA	E		98.9%	911 cps

**Table 3:** Composition of tested matrices.

Matrix Signature	UDMA Content (wt.%)	TEGDMA Content (wt.%)	Bis-EMA Content (wt. %)	Bis-GMA Content (wt.%)
U/T 80/20	80	20	---	---
U/T 70/30	70	30	---	---
U/T 60/40	60	40	---	---
U/T 50/50	50	50	---	---
E/T 80/20	---	20	80	---
U/E/T 70/10/20	70	20	10	---
U/E/T 60/20/20	60	20	20	---
U/E/T 50/30/20	50	20	30	---
U/E/T 40/40/20	40	20	40	---
G/T 80/20	---	20	---	80
U/G/T 70/10/20	70	20	---	10
U/G/T 60/20/20	60	20	---	20
U/G/T 50/30/20	50	20	---	30
U/G/T 40/40/20	40	20	---	40

**Table 4:** The mean values of the water sorption, after 120 days, and dissolution (weight loss, absolute value) of the tested matrices with standard deviations (SD).

	Sorption after 120 Days (wt.%)	SD	Dissolution (wt.%)	SD
U/T 80/20	3.3092	0.1843	0.3465	0.1336
U/T 70/30	3.7575	0.2674	0.4832	0.0759
U/T 60/40	4.0989	0.1561	0.4488	0.0221
U/T 50/50	4.0548	0.0803	0.3997	0.0580
E/T 80/20	1.1094	0.0916	0.1646	0.0896
U/E/T 70/10/20	2.7707	0.0089	0.5118	0.0775
U/E/T 60/20/20	2.5219	0.0562	0.4014	0.0775
U/E/T 50/30/20	2.3936	0.0703	0.3160	0.1450
U/E/T 40/40/20	1.8683	0.0778	0.3243	0.0796
G/T 80/20	2.9333	0.1986	0.5991	0.0759
U/G/T 70/10/20	3.0348	0.1340	0.6208	0.1615
U/G/T 60/20/20	2.8643	0.0728	0.5860	0.0851
U/G/T 50/30/20	2.9782	0.2060	0.6064	0.1655
U/G/T 40/40/20	2.8182	0.1324	0.6192	0.0604

## Data Availability

Data available in a publicly accessible repository Zenodo at 10.5281/zenodo.4772497, https://zenodo.org/record/4772497#.YKaRMrUzZPY.
